# Adaptogenic and Neuroprotective Effects of the Thai Herbal Formula AYW-KK-04 Against Chronic Stress-Induced Cognitive Impairment

**DOI:** 10.3390/ph19020339

**Published:** 2026-02-21

**Authors:** Pathomporn Saisud, Orawan Monthakantirat, Prathan Luecha, Suppachai Tiyaworanant, Abdulwaris Mading, Yutthana Chotritthirong, Sunanthra Ruangrit, Nawarat Jintanamaneerat, Jarurat Trakanchan, Juthamart Maneenet, Suresh Awale, Yaowared Sumanont

**Affiliations:** 1 Graduate School of Pharmaceutical Sciences, Khon Kaen University, Khon Kaen 40002, Thailand; patthomporn.s@kkumail.com (P.S.); abdulwaris.m@kkumail.com (A.M.); yutthana_ch@kkumail.com (Y.C.); sunansa.r@kkumail.com (S.R.); 2 Division of Pharmaceutical Chemistry, Faculty of Pharmaceutical Sciences, Khon Kaen University, Khon Kaen 40002, Thailand; oramon@kku.ac.th (O.M.); nawaratji@kkumail.com (N.J.); jarurat_t@kkumail.com (J.T.); 3 Division of Pharmacognosy and Toxicology, Faculty of Pharmaceutical Sciences, Khon Kaen University, Khon Kaen 40002, Thailand; prathanl@kku.ac.th (P.L.); suptiy@kku.ac.th (S.T.); 4 Natural Drug Discovery Laboratory, Institute of Natural Medicine, University of Toyama, 2630 Sugitani, Toyama 930-0194, Japan; juthamar@inm.u-toyama.ac.jp (J.M.); suresh@inm.u-toyama.ac.jp (S.A.)

**Keywords:** AYW-KK-04 polyherbal formulation, adaptogen, neuroprotective potential, phytochemical study, CREB/BDNF signaling pathway, Nrf2/Keap pathway, unpredictable chronic mild stress

## Abstract

**Background/Objectives:** Unpredictable chronic mild stress exposure is a primary driver of cognitive decline, largely mediated by hypothalamic–pituitary–adrenal (HPA) axis dysregulation and subsequent oxidative neurotoxicity. In traditional Thai medicine, the AYW-KK-04 formulation—a complex polyherbal remedy—has long been utilized as a “Ya Aayu-Wattana” to restore vitality and elemental balance, yet its neurobiological mechanisms remain poorly understood. This study aimed to evaluate the adaptogenic and neuroprotective potential of AYW-KK-04 against cognitive impairment. **Methods:** Unpredictable Chronic Mild Stress (UCMS)-induced cognitive impairment in a ICR mouse model. Total phenolic and flavonoid contents and antioxidant capacity (ABTS assay) of AYW-KK-04 were determined. Behavioral assessments using Y-maze test, novel object recognition test (NORT), and Morris Water Maze (MWM) test. BDNF, CREB, Nrf and Keap1 mRNA gene expression, SOD and CAT enzymatic activity and lipid peroxidation assay were investigated to clarify the mechanisms of action. Moreover, HPLC chromatography was studied to quantify the active compounds of the AYW-KK-04 formulation. **Results:** It demonstrated that oral administration of AYW-KK-04 significantly reversed UCMS-induced memory deficits. At the molecular level, AYW-KK-04 effectively upregulated BDNF and CREB mRNA expression in the frontal cortex and hippocampus, suggesting a restoration of synaptic plasticity. Simultaneously, the formulation activated the Nrf2/Keap1 signaling pathway, leading to enhanced SOD and CAT enzymatic activities and a marked reduction in MDA-mediated lipid peroxidation. HPLC analysis confirmed the presence and consistency of key bioactive constituents. **Conclusions:** These findings suggest that the adaptogenic properties of AYW-KK-04 arise from its dual capacity to reinforce neurotrophic support and bolster the endogenous antioxidant shield, providing a mechanistic support for the traditional use of AYW-KK-04 as an adaptogenic formulation and highlighting its potential as a multi-target intervention for stress-related cognitive dysfunction.

## 1. Introduction

Prolonged elevation of glucocorticoids (GCs), a hallmark of chronic stress exposure, exerts profound detrimental effects on hippocampal structure and function, ultimately leading to learning and memory impairment [1]. Sustained GC exposure disrupts the hypothalamic–pituitary–adrenal (HPA) axis homeostasis by hyperactivating glucocorticoid receptor (GRs) in hippocampal neurons, triggering a cascade of molecular events including suppression of brain-derived neurotrophic factor (BDNF), inhibition of neurogenesis, and impairment of long-term potentiation [2]. Importantly, excessive GC activation significantly enhances metabolic and mitochondrial burden in hippocampal neurons, resulting in heightened production of reactive oxygen species (ROS) and subsequent oxidative damage [3]. Moreover, chronic GC exposure dysregulates the nuclear factor erythroid 2-related factor 2 (Nrf2)/Kelch-like ECH-associated protein 1 (Keap1) signaling cascade, impairing the activation of Nrf2-dependent antioxidant gene expression and further weakening the cellular capacity, such as superoxide dismutase (SOD) and catalase (CAT) to counteract oxidative insults [4]. These converging mechanisms accelerate neuronal vulnerability, promote dendritic degeneration within the hippocampus, and ultimately lead to deficits in cognitive performance [5].

Enhancing the body’s ability to adapt to physiological stress and maintain internal equilibrium has become an important area of interest in herbal research and integrative health sciences [6,7]. Several plant-derived compounds are recognized as adaptogens—substances that enhance resistance to physical, chemical, or biological stressors and support the maintenance of homeostasis [8]. At the molecular level, adaptogens exert their effects through multiple mechanisms, including modulation of the HPA axis, regulation of immune responses, reduction in neuroinflammation, reinforcement of endogenous antioxidant systems, and preservation of neuronal integrity [8,9]. These modern concepts parallel the principles of traditional Thai medicine, which emphasize the balance of the three fundamental elements, *Tri-dhatu*—*Vata*, *Pitta*, and *Sleshma*. Disruption of this balance is traditionally associated with stress-induced deterioration of physiological and mental functions [10]. The restoration of elemental harmony forms the basis of traditional rejuvenating remedies known as “Ya Aayu-Wattana” [10,11]. AYW-KK-04 is derived from this concept, providing an ethnopharmacological rationale for evaluating its adaptogenic and neuroprotective effects against chronic stress-induced cognitive impairment.

The AYW-KK-04 formulation was developed based on these principles by selecting herbal components known to support elemental balance, strengthen the body, and contribute to antioxidative and immunomodulatory activity. AYW-KK-04 consists of 22 herbal ingredients as shown in Table 1, most of which are listed in the Thai National List of Essential Medicines for traditional remedies. A key component of the formulation is Triphala—a combination of *Terminalia chebula*, *Terminalia bellirica*, and *Phyllanthus emblica*—which accounts for approximately 26.5% of the formulation. Experimental studies in animal models have shown that Triphala exhibits strong anti-stress and antioxidative effects, including the reduction in lipid peroxidase (LPO) levels, an important indicator of oxidative damage [12,13,14]. Additionally, individual constituents of Triphala demonstrate significant neuroprotective and cognitive-enhancing properties; *Phyllanthus emblica* has been reported to exert antioxidant and neuroprotective effects on neuronal integrityand function [15], and *Terminalia chebula* has been shown to alleviate cognitive impairment through modulation of oxidative stress and neuroinflammation [16]. Moreover, aqueous extract of *Terminalia bellirica* fruit has been observed to improve cognitive performance in rodent models, potentially via its antioxidant and neuroprotective actions [17]. Other key component in the formulation is Trikatu-a combination of *Zingiber officinale* Roscoe., *Piper retrofractum* Vahl., and *Piper nigrum* L., which accounts for approximately 16.07% of the remedy, has also been reported to possess antioxidant properties [18,19].

These components collectively suggest that AYW-KK-04 may exert broad biological activity through the reduction in reactive oxygen species (ROS), the enhancement of endogenous antioxidant enzyme systems, the protection of cellular structures from oxidative injury, and the support of fundamental physiological energy processes. Such mechanisms closely align with the established characteristics of adaptogens, which help improve the body’s resilience to stress and promote recovery. Given its composition and traditional uses, AYW-KK-04 presents promising potential as a health-supporting herbal formulation with dual adaptogenic and antioxidant properties. As there is currently a lack of scientifically substantiated evidence on the neuropharmacological effects of AYW-KK-04, this study aims to clarify the impact of AYW-KK-04 on UCMS-induced cognitive impairment and to elucidate a potential mechanism underlying this effect. Additionally, phytochemical profiling of AYW-KK-04 has been conducted using high-performance liquid chromatography (HPLC), enabling the identification and quantification of key bioactive constituents within the formulation.

## 2. Results

### 2.1. Total Phenolic and Total Flavonoids Contents in AYW-KK-04 Formula and Its Herbal Components

Phenolic compounds and flavonoids derived from medicinal plants are widely recognized for their diverse pharmacological activities, particularly their antioxidant and neuroprotective effects [20]. To investigate the association between these bioactive phytochemicals and the observed biological effects of the AYW-KK-04 formulation, the total phenolic content (TPC) and total flavonoid content (TFC) of ethanolic extracts from AYW-KK-04 and each of its herbal ingredients were systematically determined. The results are presented in Table 2.

### 2.2. Effect of AYW-KK-04 Formula and Its Herbal Components on Antioxidant Activity

The antioxidant potential of the AYW-KK-04 formulation, along with its constituent herbal ingredients derived from 22 medicinal plants, was evaluated using the 2,2′ azinobis(3-ethylbenzothiazoline-6-sulfonic acid) (ABTS) radical scavenging assay. The results demonstrated that AYW-KK-04 possessed notable antioxidant activity, with an IC_50_ value of 5.98 ± 0.69 µg/mL. In addition, the IC_50_ values representing the antioxidant capacities of the individual medicinal plants included in the formulation are summarized in Table 2.

### 2.3. Effect of the AYW-KK-04 Formula on UCMS-Induced Cognitive-like Behavior

**Figure 1:**
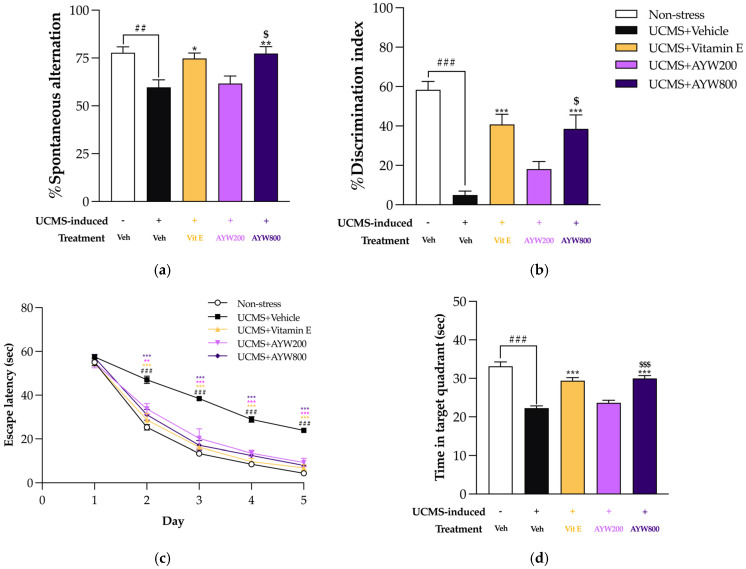
The effect of AYWKK-04 and vitamin E on the UCMS-induced cognitive impairment in the (**a**) Y-maze test, (**b**) NORT, and (**c**) Morris water maze (MWM), training phase session, and (**d**) probe test session. Each column represents the mean ± SEM (*n* = 10). ^##^ *p* < 0.01, ^###^ *p* < 0.001 compared to the vehicle-treated non-stress group. * *p* < 0.05, ** *p* < 0.01, *** *p* < 0.001 compared to the vehicle-treated UCMS group. ^$^ *p* < 0.05, ^$$$^ *p* < 0.001 compared with different doses of the AYW-KK-04.

The neuroprotective potential of the AYW-KK-04 formulation against cognitive impairments induced by unpredictable chronic mild stress (UCMS) was evaluated using a battery of behavioral assessments, including the Y-maze, Novel Object Recognition test (NORT), and Morris Water Maze (MWM). Spatial and non-spatial working memory were assessed using the Y-maze and NORT, respectively, while spatial reference memory was examined through the MWM task. Results from the Y-maze revealed that mice exposed to UCMS exhibited marked cognitive deficits, as indicated by a significant reduction in spontaneous alternation behavior compared with non-stressed controls. Administration of AYW-KK-04, particularly at a dose of 800 mg/kg/day, as well as vitamin E (100 mg/kg/day), effectively ameliorated these impairments. Consistently, in the NORT, UCMS mice receiving vehicle treatment were unable to distinguish between familiar and novel objects, reflecting compromised non-spatial working memory. In contrast, treatment with vitamin E or AYW-KK-04 at 800 mg/kg/day significantly improved object discrimination performance (Figure 1a,b) (Supplement S1 and S2).

In the MWM test, although all groups demonstrated learning during training, UCMS-exposed mice exhibited prolonged escape latencies and reduced time spent in the target quadrant during the probe test compared with non-stressed controls, indicating deficits in both memory acquisition and retrieval. These impairments were significantly attenuated by repeated treatment with vitamin E or AYW-KK-04 (800 mg/kg/day) (Figure 1c,d) (Supplement S3).

### 2.4. Effect of the AYW-KK-04 on the Nrf2-Keap1 and BDNF Pathways in UCMS-Exposed Mice

**Figure 2:**
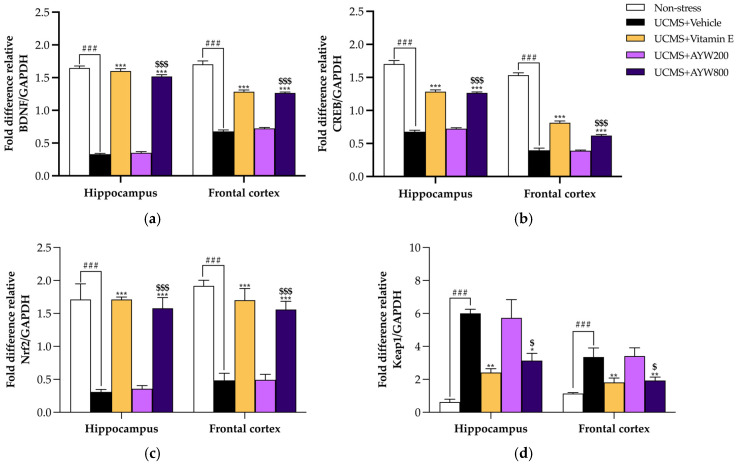
Effect of the AYW-KK-04 formula on the Nrf2-Keap1 Pathway in UCMS-induced Mice Brains, (**a**) BDNF mRNA expression, (**b**) CREB mRNA expression, (**c**) Nrf2 mRNA expression, and (**d**) Keap1 mRNA expression. Each column represents the mean ± SEM (*n* = 5). ^###^ *p* < 0.001 vs. the non-stress group. * *p* < 0.05, ** *p* < 0.01, *** *p* < 0.001 vs. the UCMS-vehicle group. ^$^ *p* < 0.05, ^$$$^ *p* < 0.001 between AYW-KK-04 treatment.

Quantitative real-time PCR (qPCR) was employed to evaluate the transcriptional levels of genes related to neuroplasticity and oxidative stress regulation, including BDNF, CREB, Nrf2, and Keap1, in the frontal cortex and hippocampus of mouse brains. As illustrated in Figure 2, vehicle-treated UCMS mice exhibited a significant reduction in BDNF and CREB mRNA expression in both brain regions compared with non-stressed control mice. In contrast, daily administration of the AYW-KK-04 formulation at a dose of 800 mg/kg or vitamin E markedly restored gene expression, resulting in significant upregulation of BDNF and CREB transcripts in the frontal cortex and hippocampus (Figure 2a,b).

To elucidate the molecular basis of the antioxidant activity of AYW-KK-04, the transcript levels of Nrf2 and Keap1 were initially analyzed using real-time PCR. As presented in Figure 2c,d, mice subjected to UCMS and receiving vehicle treatment displayed a marked suppression of Nrf2 mRNA expression, concomitant with a significant elevation of Keap1 transcripts in both the hippocampus and frontal cortex when compared with the non-stressed control group. Notably, treatment with 800 mg/kg AYW-KK-04 formula or vitamin E effectively attenuated these effects by significantly upregulating Nrf2 mRNA and modulating Keap1 expression. These results suggest that 800 mg/kg/day AYW-KK-04 formula or vitamin E can activate the Nrf2/Keap1 signaling pathway, potentially enhancing the cellular defense against chronic stress-induced oxidative damage.

### 2.5. Effect of the AYW-KK-04 Formula on Oxidative Stress and Antioxidant Enzyme Activities in the Brains

**Figure 3:**
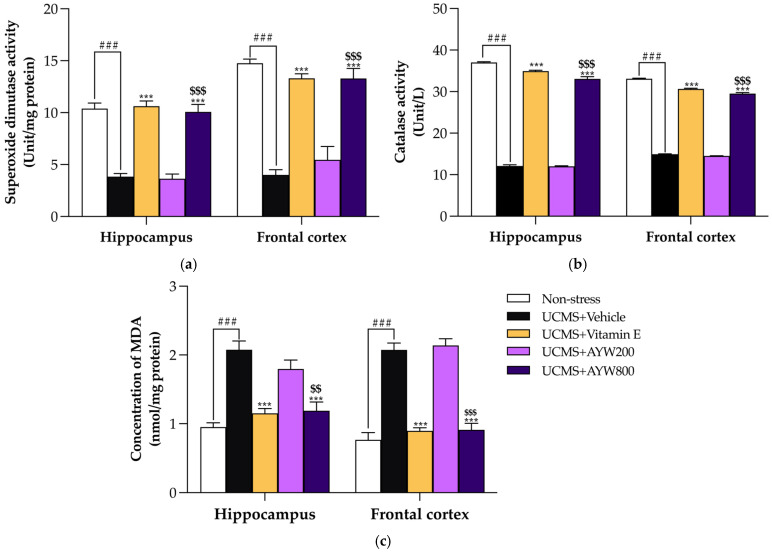
Effect of the AYW-KK-04 formula on Antioxidant Enzyme Activities and lipid peroxidation in hippocampus and frontal cortex, (**a**) superoxide dismutase (SOD) activity, (**b**) catalase (CAT) activity and concentration of MDA (**c**). Each column represents the mean ± SEM (*n* = 5). ^###^ *p* < 0.001 vs. the non-stress group. *** *p* < 0.001 vs. the UCMS-vehicle group. ^$$^ *p* < 0.01, ^$$$^ *p* < 0.001 between AYW-KK-04 treatment.

In line with the transcriptional activation of the Nrf2 signaling pathway, the functional activities of the antioxidant enzymes superoxide dismutase (SOD) and catalase (CAT) were subsequently assessed. Prolonged exposure to UCMS resulted in a significant decline in SOD and CAT activities in both the hippocampus and frontal cortex when compared with non-stressed controls. Conversely, treatment with the AYW-KK-04 formulation at a dose of 800 mg/kg/day or with vitamin E markedly enhanced the activities of these enzymes relative to the UCMS plus vehicle group (*** *p* < 0.001) (Figure 3a,b).

To further verify whether the reinforcement of the antioxidant defense system was associated with a reduction in oxidative injury, levels of malondialdehyde (MDA), a widely recognized indicator of lipid peroxidation, were quantified. As illustrated in Figure 3c, mice exposed to UCMS displayed a pronounced elevation in MDA levels relative to non-stressed controls, reflecting substantial oxidative stress. In contrast, administration of the AYW-KK-04 formulation at 800 mg/kg/day or vitamin E significantly attenuated MDA concentrations in both the hippocampus and frontal cortex when compared with the UCMS plus vehicle group (*** *p* < 0.001). Collectively, these results indicate that the AYW-KK-04 formula confers effective protection against chronic stress-induced oxidative damage by modulating the Nrf2/Keap1 signaling pathway, enhancing antioxidant enzyme activities, and consequently limiting lipid peroxidation (Supplement S4–S6).

### 2.6. HPLC Analysis of the Constituents of the AYW-KK-04 Extracts and the Validation Method

**Figure 4:**
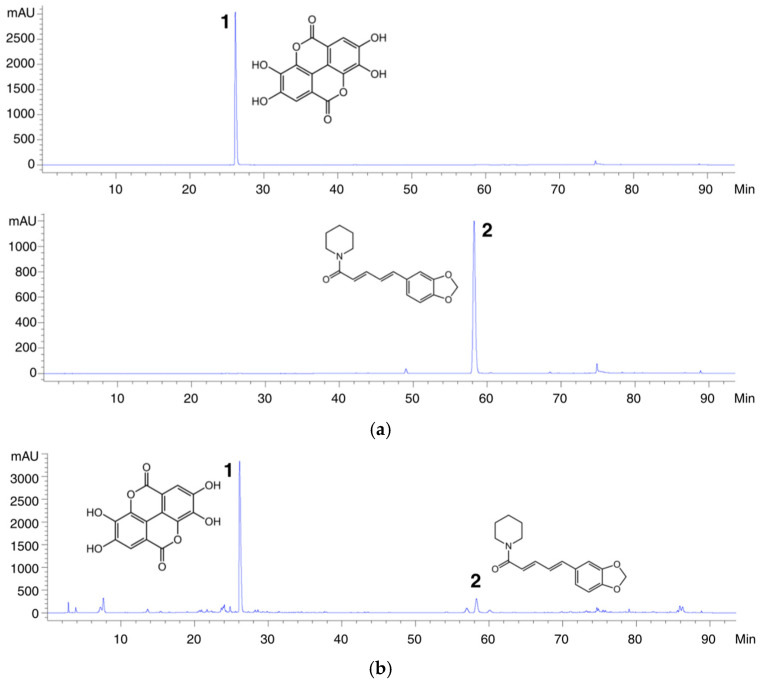
HPLC Chromatogram of (**a**) standard: ellagic acid (**1**), and piperine (**2**), and (**b**) the AYW-KK-04 extract.

The herbal formulation comprises several medicinal plants that are rich in bioactive phytochemicals with well-documented pharmacological activities. *Phyllanthus emblica* Linn., *Terminalia bellirica* (Gaertn.) Roxb., and *Terminalia chebula* Retz. var. *chebula*, which together constitute the Triphala remedy, are prominent sources of polyphenolic compound, ellagic acid known for its strong antioxidant and neuroprotective properties. Piperine, the principal alkaloid found in *Piper retrofractum*, is known to enhance bioavailability of co-administered compounds and to exert anti-inflammatory and antioxidant activities. Accordingly, ellagic acid and piperine were selected as chemical marker compounds for the HPLC characterization of the AYW-KK-04 formulation. Based on the method validation results, the established analytical procedure was demonstrated to be robust and appropriate for the quantitative determination of both compounds. Intra-day and inter-day precision analyses yielded percentage relative standard deviation (%RSD) values below 2%, while excellent linearity was observed, as evidenced by a strong correlation between peak area and analyte concentration (R^2^ > 0.999). Method accuracy, evaluated through recovery experiments using spiked standards, consistently ranged from 99% to 105%. Collectively, these validation parameters confirm that the proposed HPLC method is reliable and suitable for the analysis of ellagic acid and piperine. Representative HPLC chromatograms of standard ellagic acid (**1**) and piperine (**2**) are presented in Figure 4.

HPLC analysis was subsequently conducted to quantify the selected marker compounds in the AYW-KK-04 formulation. As illustrated in Figure 4, the observed retention times (ellagic acid; 26.124, and piperine; 58.224) corresponded closely with those obtained from the reference standards. Quantitative assessment revealed the presence of two major constituents in the formulation, namely ellagic acid at a concentration of 3.516 ± 0.020 mg/g of powder and piperine at 0.051 ± 0.010 mg/g of powder.

## 3. Discussion

The findings of this study indicate that AYW-KK-04 markedly alleviates cognitive impairments caused by Unpredictable Chronic Mild Stress (UCMS), as reflected by enhanced performance in the Y-maze, Novel Object Recognition, and Morris Water Maze tasks. These behavioral benefits may be attributed to the synergistic effects of bioactive compounds present in the formulation, which are characterized by substantial total flavonoid and phenolic contents and were confirmed through HPLC analysis. The pronounced antioxidant capacity observed in vitro further substantiates the adaptogenic properties of AYW-KK-04. Moreover, administration of AYW-KK-04 in the UCMS mouse model contributes to the restoration of redox homeostasis by mitigating excessive reactive oxygen species generation and reducing lipid peroxidation in both the hippocampus and frontal cortex. These neuroprotective effects appear to be mediated by the upregulation of CREB and BDNF gene expression, along with the regulation of the Nrf2/Keap1 signaling pathway, which collectively enhance the activities of key antioxidant enzymes, including superoxide dismutase and catalase, thereby counteracting oxidative stress associated with hyperactivation of the hypothalamic–pituitary–adrenal (HPA) axis.

The robust neuroprotective and adaptogenic effects of AYW-KK-04 observed in this study can be attributed to its rich phytochemical profile. Our analysis revealed high TPC and TFC contents, which directly correlate with the potent free-radical scavenging activities observed in the ABTS assays (Table 2). Polyphenolic compounds are known to neutralize reactive oxygen species directly and to activate endogenous antioxidant defense systems [21], suggesting that the observed bioactivity of AYW-KK-04 may be closely linked to its phytochemical composition. Phytochemical investigation was further substantiated by high performance liquid chromatography (HPLC) analysis through the identification and quantification of ellagic acid (**1**) and piperine (**2**) as constituents of the formulation. The HPLC method was fully validated, demonstrating acceptable linearity, precision, accuracy, and sensitivity, thereby ensuring analytical reliability and reproducibility of the extract. Standardization of these marker compounds provides a robust chemical basis for the observed antioxidant and neuroprotective effects and strengthens the translational relevance of the formulation. Ellagic acid (**1**) is well recognized for its potent free radical–scavenging and neuroprotective properties, while piperine (**2**) is known to modulate oxidative stress and enhance the bioavailability of co-administered phytochemicals [20,22]. The identification and quantification of key bioactive compounds strengthen the scientific rigor of this study and support the observed biological effects. The combination of multiple phytochemicals with complementary antioxidant and neuroprotective activities likely underlies the formulation’s efficacy, emphasizing the importance of synergistic interactions inherent to traditional multi-herbal remedies as similar as in our previous study, Suk-SaiYasna, a remedy composed of multiple phytochemicals including delta-9-tetrahydrocannabinol, delta-9-tetrahydrocannabinolic acid A, gallic acid, myricetin, piperine, and gingerol, alleviated UCMS-induced cognitive dysfunction through antioxidant properties and modulation of multiple pathways [23].

Behavioral evaluations further revealed that exposure to UCMS induced pronounced impairments in several aspects of hippocampus-dependent cognitive function. These deficits were reflected by decreased spontaneous alternation in the Y-maze, lower discrimination indices in the Novel Object Recognition Test (NORT), and increased escape latency accompanied by diminished target quadrant preference in the Morris Water Maze (MWM). Such observations agree with earlier studies demonstrating that UCMS adversely affects learning ability, memory formation, and spatial navigation processes [23,24]. In the UCMS in vivo model, chronic stress is characteristically associated with impaired hippocampal neurogenesis and synaptic plasticity, primarily resulting from the downregulation of brain-derived neurotrophic factor (BDNF) and its upstream transcriptional regulator, cAMP response element-binding protein (CREB) [25,26]. Consistent with this mechanism, the present findings show that treatment with AYW-KK-04 markedly increased CREB and BDNF mRNA expression in both the frontal cortex and hippocampus. This molecular modulation provides a mechanistic basis for the observed improvements in cognitive performance across the Y-maze, NORT, and MWM tasks. Restoration of BDNF signaling may facilitate synaptic strengthening, including long-term potentiation (LTP) and dendritic arborization, which are critical for spatial learning and object recognition memory [27]. Notably, these neurobiological effects are in line with the traditional “Ya Aayu-Wattana” rejuvenation concept, which emphasizes the synergistic use of herbal formulations to support neural function and correct stress-induced elemental imbalance (Tri-dhatu) arising from prolonged environmental challenges.

The adaptogenic properties of AYW-KK-04 are further elucidated by its impact on the Nrf2/Keap1 signaling pathway. Under chronic UCMS-induced stress, the excessive production of ROS typically leads to the degradation of Nrf2, weakening the cell’s endogenous defense [28]. Our study showed that AYW-KK-04 effectively modulated this pathway by upregulating Nrf2 and downregulating Keap1 mRNA expression. This signaling activation is essential, as Nrf2 functions as a central transcriptional regulator that interacts with the Antioxidant Response Element (ARE) to initiate the expression of phase II detoxifying and antioxidant enzymes [29]. The elevation in superoxide dismutase (SOD) and catalase (CAT) activities observed in this study provides compelling evidence of this downstream regulatory effect. Functionally, SOD constitutes the primary enzymatic defense by catalyzing the conversion of superoxide anions into hydrogen peroxide, which is subsequently decomposed into water and oxygen by CAT [30]. The synergy between these enzymes, bolstered by the constituents of the formulation, creates a comprehensive shield against oxidative insults in the brain’s most vulnerable regions [31]. The ultimate success of the AYW-KK-04 intervention is reflected in the significant reduction in MDA levels within the frontal cortex and hippocampus. MDA is a toxic end-product of lipid peroxidation that disrupts cell membrane fluidity and integrity. By neutralizing ROS through both direct (phytochemical scavenging) and indirect (Nrf2-mediated enzyme induction) mechanisms [32], AYW-KK-04 prevents the oxidative degradation of neuronal lipids.

The safety of herbal formulations is a critical consideration, particularly when relatively high doses are employed in experimental studies. AYW-KK-04 formulation consists mainly of Triphala and Trikatu, which have been extensively used for centuries and are well reported for their safety. No acute toxicities were observed when 5000 mg/kg of TPL was given orally to Sprague Dawley rats. For the chronic toxicity study, TPL was administered at doses of 600, 1200, and 2400 mg/kg/day for 270 days. Researchers monitored the rats’ general health, behavior, and body and organ weights [33]. Trikatu demonstrated safety in mice in acute toxicity tests at 2000 mg/kg/day and in subacute studies at 5, 50, and 300 mg/kg/day for 28 days. No significant changes were observed in mortality, morbidity, gross pathology, weight gain, vital organ weight, hematological parameters, or biochemical parameters, including serum and lipid profiles, in either acute or subacute toxicity studies. Therefore, a high dose of the AYW-KK-04 formulation containing Triphala at 212 mg/kg/day and Trikatu at 128 mg/kg/day was considered safe [34]. The existing literatures support the safety of the AYW-KK-04 formulation. In addition, dose selection was guided by human equivalent dose calculations and supported by the absence of observable toxicity during the experimental period.

Finally, the comprehensive neuroprotective and adaptogenic effects of AYW-KK-04 observed in this study can be attributed to the sophisticated synergy between its two primary herbal clusters: Triphala and Trikatu, with the minor component herbs. Our HPLC analysis confirmed the presence of key bioactive markers, particularly from the Triphala component, which constitutes a significant 26.7% of the formulation. Specifically, *Phyllanthus emblica* (10.5%), *Terminalia bellirica* (9%), and *Terminalia chebula* (7.2%) are rich sources of hydrolyzable tannins, such as ellagic acid (**1**), as shown in the HPLC analysis. Crucially, the integration of the Trikatu cluster—comprising *Piper retrofractum* (7.21%), *Zingiber officinale* (7.21%), and *Piper nigrum* (1.5%)—represents a strategic application of traditional Thai medical wisdom. In the Thai tradition, this ‘pungent-hot’ formula is specifically used to balance the Vata (wind) element, which is often dysregulated during periods of physiological stress. From a modern pharmacological standpoint, our HPLC quantification of piperine (**2**) highlights a key mechanism of AYW-KK-04 as a bio-enhancer. Piperine (**2**) is well-documented for its ability to increase the bioavailability of polyphenols by modulating metabolic pathways and enhancing intestinal permeability [35]. Therefore, the presence of the Trikatu cluster likely facilitates the delivery of the Triphala-derived antioxidants and other bioactive constituents to the frontal cortex and hippocampus. This ensures sustained activation of the BDNF/CREB and Nrf2/Keap1 pathways, ultimately leading to the enhanced enzymatic activities of SOD and CAT and the significant suppression of MDA-mediated lipid peroxidation observed in our UCMS model.

This multi-layered defense mechanism of AYW-KK-04, a traditional Thai polyherbal, satisfies the modern criteria for an adaptogen: it non-specifically increases the body’s resilience to stress and helps maintain homeostatic equilibrium (Vata, Pitta, and Sleshma balance) even under severe physiological pressure. Taken together, these present findings underscore the potential of AYW-KK-04 as a high-efficacy adaptogenic remedy for stress-related cognitive disorders.

## 4. Materials and Methods

### 4.1. Plant Materials and AYW-KK-04 Preparation

The botanical materials used to prepare the AYW-KK-04 formulation were obtained and processed at the Faculty of Pharmaceutical Sciences, Khon Kaen University, Khon Kaen, Thailand. The formulation consists of 22 medicinal plant species, the details of which are listed in Table 1. Botanical authentication of all plant materials was conducted by Suppachai Tiyaworanant, Director of the Pharmacy Museum, Faculty of Pharmaceutical Sciences, Khon Kaen University. Voucher specimens were prepared and permanently deposited in the herbarium of the Faculty of Pharmaceutical Sciences, Khon Kaen University to ensure future reference and taxonomic verification.

Before extraction, all medicinal plants were washed, chopped, oven-dried at 50 °C, and ground into fine powders. The dried plant powders were accurately weighed and mixed in accordance with the formulation ratio of Triphala 26.5% and Trikatu 16.07% of the remedy. Individual plant powders, as well as the combined AYW-KK-04 formulation, was extracted by maceration using 95% ethanol at room temperature for 72 h, with the extraction cycle performed three times. The resulting extracts were filtered through Whatman No. 1 filter paper and concentrated under reduced pressure using a rotary evaporator at 50 °C. Extraction yields were quantified on a weight-to-weight basis, and the concentrated extracts were stored at −20 °C pending further analysis.

### 4.2. Determination of Total Phenolic and Total Flavonoid Contents

The total phenolic content (TPC) of the ethanolic extracts from AYW-KK-04 and its individual medicinal plant components was quantified using the Folin–Ciocalteu colorimetric method. Briefly, each extract was prepared in ethanol, and a 20 µL aliquot was reacted with 100 µL of Folin–Ciocalteu reagent. Following a 5 min reaction, 80 µL of 7% (*w*/*v*) sodium carbonate was added, and the mixture was incubated for 30 min at room temperature. Absorbance was subsequently measured at 760 nm. The TPC was quantified using a gallic acid (GA) standard curve and expressed as mg GAE/g extract. All analyses were performed in triplicate [36].

Total flavonoid content (TFC) was determined using an aluminum chloride colorimetric assay. Brief, a 20 µL aliquot of each extract was reacted with 15 µL of 2.5% aluminum chloride solution, 20 µL of sodium acetate (100 g/L), and 145 µL of distilled water in a 96-well microplate. Following a 15 min incubation at ambient temperature, absorbance was subsequently read at 430 nm. TFC values were calculated using a quercetin standard curve and reported as mg QE/g extract. Each sample was assayed in triplicate [36].

### 4.3. In Vitro Antioxidant Activity by ABTS Assay

The ABTS radical cation decolorization method was employed to assess the antioxidant capacity of the AYW-KK-04 extract. A stable ABTS•^+^ solution was prepared by incubating 7 mM ABTS with 2.45 mM potassium persulfate for 12–16 h in the dark at room temperature. Subsequently, 50 µL of extract was combined with 100 µL of ABTS•^+^ and incubated for 15 min at ambient temperature. Absorbance reductions were recorded at 734 nm, and Trolox was used as the calibration standard for activity calculations [36,37].

### 4.4. Animals

Fifty male Institute of Cancer Research (ICR) mice (5 weeks old, 20–30 g), were sourced from Nomura Siam International Co., Ltd., Bangkok, Thailand and housed under controlled conditions at the Laboratory Animal Unit, Faculty of Pharmaceutical Sciences, Khon Kaen University. Animals were maintained at 22 ± 2 °C and 45 ± 2% relative humidity on a 12 h light/dark cycle (lights on from 06:00 to 18:00) with free access to food and water. All procedures were approved by the Animal Ethics Committee of Khon Kaen University (IACUC-KKU-46/66) and complied with NIH guidelines (NIH Publication No. 80-23, revised 2011) and ARRIVE 2.0 standards.

### 4.5. Unpredictable Chronic Mild Stress (UCMS) Paradigm

**Figure 5:**
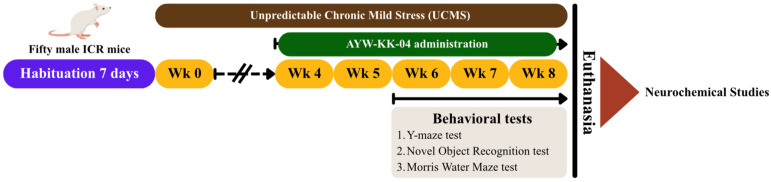
Schematic drawing of unpredictable chronic mild stress (UCMS) procedure.

As illustrated in Figure 5, mice were randomly assigned to five experimental groups. Animals in the control group were maintained under standard housing conditions without exposure to stress, whereas the remaining groups were subjected to an unpredictable chronic mild stress (UCMS) paradigm for one week prior to treatment, following a previously established protocol with minor modifications [38]. The UCMS procedure consisted of a series of mild stressors administered in a random order to prevent habituation.

Stressors were categorized according to their frequency of application. Twice-weekly stressors included housing in a cage tilted at a 45° angle for 12 h, restricted food access (five small pellets for 1 h), exposure to an empty water bottle for 3 h, continuous illumination for 36 h, and exposure to intermittent noise for 3 or 5 h. Additional stressors administered once weekly included housing in a wet cage for 21 h, combined food and water deprivation for 18 h, and temporary pairing of mice from different cages for 2 h. All stress procedures commenced during the first week and were repeatedly applied throughout the experimental period to induce a persistent chronic stress condition.

### 4.6. Experimental Design and Drug Administration

The experimental design was adapted from a previously published protocol [38]. Mice were randomly assigned into five groups (*n* = 10 per group). The first group served as the non-stressed control and received vehicle treatment consisting of 0.5% sodium carboxymethyl cellulose (SCMC; Sigma-Aldrich^®^, St. Louis, MO, USA) at a dose of 1 mL/kg via oral administration. The second group comprised UCMS-exposed mice treated with the same vehicle. A positive control group included UCMS-exposed mice administered α-tocopherol (vitamin E; Sigma-Aldrich^®^, St. Louis, MO, USA) suspended in 0.5% SCMC at a dose of 100 mg/kg, p.o. The remaining two experimental groups consisted of UCMS-induced mice treated orally with the AYW-KK-04 formulation at doses of 200 or 800 mg/kg. The selected doses of AYW-KK-04 were determined based on human equivalent dose (HED) calculations. AYK-KK-04 was freshly prepared daily as a suspension in 0.5% SCMC. The required amount of AYK-KK-04 powder was accurately weighed and gradually dispersed in the vehicle with continuous stirring to ensure uniform suspension before administration. All treatments were orally administered once daily via oral gavage. Treatment administration began during the fourth week of the experimental timeline and was continued daily in parallel with UCMS exposure. Behavioral assessments were carried out in the sixth week, with treatments administered 1 h prior to each behavioral test on the designated testing days. At the conclusion of the experimental period, mice were euthanized by intraperitoneal injection of thiopental sodium (60 mg/kg; Anesthal^®^, JAGSONPAL Pharmaceutical LTD, New Delhi, India). Blood samples were subsequently collected, and brain regions including the frontal cortex and hippocampus, along with other relevant organs, were rapidly excised and stored at −80 °C for subsequent neurochemical and molecular analyses.

### 4.7. Cognitive Performance Assessment

Spatial and working memory were assessed using three behavioral paradigms adapted from established protocols [24]. The Y-maze test evaluated hippocampus-dependent spatial memory using a three-arm apparatus constructed from black polyethylene. Each arm measured 3.8 cm at the base, 12.5 cm at the top, 40.4 cm in length, and 18.9 cm in height, with arms positioned at 60° angles. Mice were placed in one arm and allowed to explore freely for 5 min, during which arm entries were recorded. To prevent the influence of olfactory cues, the apparatus was cleaned with 70% ethanol between sessions. Spontaneous alternation was calculated according to the following equation:

%Spontaneous alternation = [(Number of alternations)/(Total arm entries − 2)] × 100(1)

The Novel Object Recognition Test (NORT) was used to assess working memory in a square open-field arena measuring 50 × 50 × 50 cm. Mice were first allowed to explore the empty arena freely for 15 min to habituate to the environment. During the sample phase, animals were introduced to two identical objects and permitted to explore for 5 min. After a 30 min retention interval, one of the familiar objects was replaced with a novel object, and exploratory behavior was recorded during a 5 min test session. To eliminate olfactory cues, the arena and all objects were thoroughly cleaned with 70% ethanol between trials. Preference for the novel object was expressed as a discrimination index, calculated as follows:

%Discrimination index = [(TN − TF)/(TN + TF)] × 100(2)

While TN and TF represent exploration times for novel and familiar objects, respectively

The Morris Water Maze (MWM) test evaluated spatial learning and memory using a circular black pool divided into four quadrants containing a submerged platform. During the acquisition phase, mice completed four daily trials over five consecutive days, starting from different quadrants positions and swimming to locate the fixed platform. Each successful trial concluded with 10 s on the platform, followed by 60 s in an isolated chamber. Escape latency was recorded and averaged across daily trials. A probe trial conducted after training removal assessed memory consolidation by measuring time spent in the target quadrant relative to that in other quadrants during a 60 s platform-free swim.

### 4.8. Measurement of Lipid Peroxidation Using TBARs Assay

Lipid peroxidation is a major contributor to oxidative injury, particularly in neural tissues, where reactive oxygen species readily attack membrane phospholipids. Malondialdehyde (MDA), a stable end product of polyunsaturated fatty acid peroxidation, is widely used as an indicator of oxidative stress and can be quantified using the thiobarbituric acid reactive substances (TBARS) assay. In the present study, MDA levels in the hippocampus and frontal cortex were measured following the protocol described by Sultana et al. [39]. Briefly, brain tissues were homogenized and mixed with trichloroacetic acid (TCA; Sigma-Aldrich^®^, St. Louis, MO, USA), followed by centrifugation at 8000× *g* for 10 min at 4 °C to remove precipitated proteins. The resulting supernatant was reacted with 0.8% thiobarbituric acid (TBA; Sigma-Aldrich^®^, St. Louis, MO, USA) and heated at 100 °C for 15 min to facilitate formation of the colored MDA–TBA adduct. Absorbance was subsequently measured at 550 nm using a spectrophotometer. MDA concentrations were quantified using a standard calibration curve and normalized to total protein content, with results expressed as nanomoles per milligram of protein.

### 4.9. Determination of Antioxidant Enzymatic Activities

Superoxide dismutase (SOD) plays a pivotal role in cellular antioxidant defense by catalyzing the dismutation of superoxide anion radicals into hydrogen peroxide and molecular oxygen, whereas catalase (CAT) further detoxifies hydrogen peroxide by converting it into water and oxygen. Accumulation of these reactive oxygen species can result in oxidative injury through damage to cellular macromolecules [40]. Thus, the coordinated actions of SOD and CAT constitute essential enzymatic mechanisms for mitigating oxidative stress. In this study, SOD and CAT activities were assessed in the hippocampus and frontal cortex. Brain tissues were homogenized in ice-cold phosphate buffer (5 mM, pH 7.4) to obtain 20% (*w*/*v*) homogenates. Enzyme activities were quantified using commercially available assay kits—the SOD Assay Kit (No. 19160) and Catalase Assay Kit (CAT100) from Sigma-Aldrich^®^ (St. Louis, MO, USA)—according to the manufacturer’s instructions. The resulting enzyme activities were normalized to total protein concentrations, which were determined using the Bradford protein assay [41].

### 4.10. Quantitative Real-Time Polymerase Chain Reaction (qRT-PCR)

The mRNA expression levels of BDNF, CREB, Nrf2, and Keap1 in hippocampal and frontal cortex tissues were quantified using quantitative real-time polymerase chain reaction (qRT-PCR). Total RNA was isolated from brain samples using TRIzol^®^ reagent (Thermo Fisher Scientific Inc., San Jose, CA, USA) following the manufacturer’s instructions. Complementary DNA (cDNA) was synthesized from the extracted RNA using oligo(dT) primers and SuperScript III reverse transcriptase (Thermo Fisher Scientific Inc., San Jose, CA, USA). Quantitative PCR was performed using qPCRBIO SyGreen Mix Separate-ROX (PCR Biosystems Ltd., London, UK) with gene-specific primers obtained from Macrogen (Seoul, Republic of Korea), as listed in Table 3. The specificity of each amplification reaction was verified by post-amplification melting curve analysis. Relative gene expression levels were calculated by normalizing target gene expression to glyceraldehyde-3-phosphate dehydrogenase (GAPDH) as the internal control and expressed as fold changes relative to the control group.

### 4.11. High-Performance Liquid Chromatography (HPLC) Analysis and the Method Validation

High-performance liquid chromatography (HPLC) was employed to quantify the marker compounds ellagic acid and piperine in the AYW-KK-04 formulation and its individual herbal constituents. For sample preparation, 1 g of AYW-KK-04 powder was extracted with 10 mL of methanol under agitation for 30 min, and the resulting extract was filtered through a 0.45 µm membrane filter before chromatographic analysis.

HPLC separation was carried out using a VDSpher PUR 100 C18-E column (250 × 4.5 mm, 5 µm) installed on an Agilent 1260 system equipped with a UV/Vis detector, with detection set at 254 nm. The mobile phase consisted of acetonitrile (solvent A) and 0.1% phosphoric acid in deionized water (solvent B), delivered at a constant flow rate of 1.0 mL/min. The gradient elution program was as follows: 5% A at 0 min, 10% at 10 min, 45% at 40 min, 50% at 60 min, 90% at 75 min, and 100% from 80 to 85 min. The injection volume for all samples was 10 µL. Each analysis was performed in triplicate to ensure precision and reproducibility.

Reference standards of ellagic acid (**1**) and piperine (**2**) were prepared prior to analysis. Serial dilutions of each standard were injected into the HPLC system to determine the limits of detection (LOD) and quantification (LOQ), defined by signal-to-noise ratios of approximately 3:1 and 10:1, respectively. Identification of ellagic acid and piperine in the samples was achieved by comparing their retention times and UV spectral profiles with those of the corresponding reference standards. Quantification was performed using external calibration curves. Standard solutions of ellagic acid (15–360 µg/mL) and piperine (50–1000 µg/mL) were prepared in methanol and analyzed by HPLC, and calibration curves were constructed by plotting mean peak areas against analyte concentrations. Method validation was conducted in accordance with the ICH guidelines (adopted 24 March 2022). Linearity was assessed by linear regression analysis, and the coefficient of determination (R^2^) was calculated from five independent measurements (*n* = 5). Accuracy was evaluated through recovery studies at three concentration levels for each standard, analyzed in five replicates. Precision was expressed as percentage relative standard deviation (%RSD) for both intra-day and inter-day variability, based on five replicate determinations.

The amounts of ellagic acid and piperine present in the samples were determined from their respective calibration equations (Y = ax + b), in which Y denotes the chromatographic peak area and X corresponds to the concentration of the analyte. The calibration parameters, including slope, intercept, coefficient of determination (R^2^), and related statistical indices, were evaluated to verify the validity and robustness of the analytical procedure.

### 4.12. Statistical Analysis

Statistical analyses were performed using paired Student’s *t*-tests to compare non-stress and stress-exposed groups. Multiple group comparisons were performed using one-way analysis of variance (ANOVA), followed by Tukey’s post hoc test for multiple group comparison. Statistical significance was defined as *p* < 0.05. Data are expressed as mean ± standard error of the mean (SEM), except for in vitro experiments, where values represent mean ± standard deviation (SD). All analyses were conducted using SigmaStat^®^ software version 3.5 (SYSTAT Software Inc., Richmond, CA, USA).

## 5. Conclusions

In conclusion, the present finding provides compelling scientific evidence for the adaptogenic and neuroprotective properties of AYW-KK-04 against UCMS-induced cognitive impairment. Our study demonstrates that the formulation effectively restores spatial learning and recognition memory by activating two critical molecular mechanisms: the BDNF/CREB pathway, which supports neuroplasticity, and the Nrf2/Keap1 signaling cascade, which reinforces the endogenous antioxidant shield. The neuroprotective effect of AYW-KK-04 is fundamentally rooted in its sophisticated polyherbal synergy, where the antioxidant-rich Triphala cluster and the bio-enhancing Trikatu cluster work in molecular mechanisms to neutralize reactive oxygen species, boost enzymatic defenses (SOD and CAT), and suppress lipid peroxidation within the frontal cortex and hippocampus. By bridging traditional Thai medical wisdom with modern pharmacological mechanisms, this study substantiates the use of AYW-KK-04 as a high-potential therapeutic strategy for mitigating the neurological consequences of chronic stress exposure.

## Figures and Tables

**Table 1:** List of medicinal plants in AYW-KK-04 remedy.

Scientific Name	Part Used	Voucher Specimen
*Phyllanthus emblica* Linn.	Fruit	KKPSH-HN-3723
*Terminalia bellirica* (Gaertn.) Roxb.	Fruit	KKPSH-HN-3724
*Terminalia chebula* Retz. var. *chebula*	Fruit	KKPSH-HN-3725
*Mesua ferrea* Linn.	Flower	KKPSH-HN-3726
*Amomum xanthioides* Wall. ex Baker	Fruit	KKPSH-HN-3727
*Brucea javanica* (L.) Merr.	Fruit	KKPSH-HN-3728
*Zingiber officinale* Roscoe.	Rhizome	KKPSH-HN-3729
*Piper retrofractum* Vahl.	Fruit	KKPSH-HN-3730
*Acorus calamus* L.	Rhizome	KKPSH-HN-3731
*Citrus aurantium* L. var. *aurantium*	Young fruit	KKPSH-HN-3732
*Citrus reticulata* Blanco	Bark	KKPSH-HN-3733
*Glycyrrhiza glabra* L.	Root	KKPSH-HN-3734
*Cyperus rotundus* L.	Rhizome	KKPSH-HN-3735
*Alpinia conchigera* Griff.	Rhizome	KKPSH-HN-3736
*Tinospora crispa* (L.)	Vine plant	KKPSH-HN-3737
*Allium sativum* L.	Rootstalk	KKPSH-HN-3738
*Piper nigrum* L.	Fruit	KKPSH-HN-3739
*Maerua siamensis* (Kurz) Pax	Root	KKPSH-HN-3740
*Cuminum cyminum* L.	Fruit	KKPSH-HN-3741
*Foeniculum vulgare* Mill. var. *dulce*	Fruit	KKPSH-HN-3742
*Pimpinella anisum* L.	Fruit	KKPSH-HN-3743
*Trachyspermum ammi* (L.) Sprague	Fruit	KKPSH-HN-3744

**Table 2:** The % yield, TPC, TFC and antioxidant activity of each medicinal herb extract in AYW-KK-04.

Scientific Name	% Yield	TPC mg GAE/g Extract	TFC mg QE/g Extract	ABTS Assay IC_50_ (ug/mL)
*Phyllanthus emblica* Linn.	21.10	237.28 ± 3.85	13.25 ± 0.08	16.93 ± 0.39
*Terminalia bellirica* (Gaertn.) Roxb.	13.63	285.67 ± 5.44	13.77 ± 0.01	9.81 ± 0.89
*Terminalia chebula* Retz. var. *chebula*	20.76	662.05 ± 8.41	28.39 ± 0.22	6.00 ± 0.02
*Mesua ferrea* Linn.	17.45	196.05 ± 0.73	18.14 ± 0.14	97.64 ± 0.23
*Amomum xanthioides* Wall. ex Baker	3.25	179.42 ± 2.75	25.62 ± 0.28	198.93 ± 10.69
*Brucea javanica* (L.) Merr.	15.58	70.87 ± 0.63	22.70 ± 0.16	1493.95 ± 2.81
*Zingiber officinale* Roscoe.	4.81	156.70 ± 1.26	24.40 ± 0.33	39.27 ± 0.15
*Piper retrofractum* Vahl.	40.97	110.42 ± 1.59	29.57 ± 0.14	329.62 ± 15.29
*Acorus calamus* L.	9.77	104.11 ± 0.36	16.07 ± 0.08	531.34 ± 0.71
*Citrus aurantium* L. var. *aurantium*	14.70	79.91 ± 0.35	25.48 ± 0.14	583.05 ± 2.74
*Citrus reticulata* Blanco	21.43	77.60 ± 0.36	15.60 ± 0.00	233.25 ± 8.77
*Glycyrrhiza glabra* L.	6.18	230.34 ± 1.31	40.20 ± 0.41	140.36 ± 9.21
*Cyperus rotundus* L.	7.08	160.07 ± 0.36	20.64 ± 0.22	197.89 ± 5.73
*Alpinia conchigera* Griff.	2.93	125.99 ± 0.35	21.39 ± 0.14	74.86 ± 1.60
*Tinospora crispa* (L.)	3.23	289.88 ± 3.84	103.92 ± 1.60	27.62 ± 0.17
*Allium sativum* L.	2.44	61.82 ± 0.35	25.43 ± 0.16	25.43 ± 0.16
*Piper nigrum* L.	7.41	156.70 ± 1.26	28.11 ± 0.16	27.66 ± 0.76
*Maerua siamensis (Kurz) Pax*	1.88	241.28 ± 0.63	145.58 ± 0.78	8.85 ± 0.92
*Cuminum cyminum* L.	7.45	522.35 ± 3.70	209.58 ± 2.79	29.02 ± 1.29
*Pimpinella anisum* L.	5.18	206.77 ± 0.96	91.36 ± 0.98	194.85 ± 2.92
*Trachyspermum ammi* (L.)	9.25	288.88 ± 1.67	120.93 ± 1.87	173.53 ± 1.23
*Foeniculum vulgare* Mill. var *dulce*	14.77	516.25 ± 3.18	156.16 ± 0.45	32.35 ± 1.33
AYW-KK-04	14.83	151.21 ± 0.09	23.41 ± 0.29	5.98 ± 0.69
Trolox (µM)		-	-	34.75 ± 1.57

**Table 3:** The primer sequences for qRT-PCR.

Genes	Primer Sequences	References
GAPDH (House-keeping gene)	**Forward:** 5′-ACCACAGTCCATGCCATCAC-3′ **Reverse:** 5′-TCCACCACCCTGTTGCTGTA-3′	[24]
BDNF	**Forward:** 5′-GACAAGGCAACTTGGCCTAC-3′ **Reverse:** 5′-CCTGTCACACACGCTCAGCTC-3′	[23]
CREB	**Forward:** 5′-TACCCAGGGAGGAGCAATAC-3′ **Reverse:** 5′-GAGGCAGCTTGAACAACAAC-3′	[23]
Nrf2	**Forward:** 5′-CAGTGCTCCTATGCGTGAA-3′ **Reverse:** 5′-GCGGCTTGAATGTTTGTC-3′	[24]
Keap1	**Forward:** 5′-CATCCACCCTAAGGTCATGGA-3′ **Reverse:** 5′-GACAGGTTGAAGAACTCCTCC-3′	[24]

## Data Availability

The original contributions presented in this study are included in the article/Supplementary Materials. Further inquiries can be directed to the corresponding author.

## Supplementary Materials

The following supporting information can be downloaded at: https://www.mdpi.com/article/10.3390/ph19020339/s1. Supplementary S1: Statistical Analysis Effect of AYW-KK-04 on UCMS-Induced Cognitive-Like Behavior using the Y-Maze Test; Supplementary S2: Statistical Analysis Effect of AYW-KK-04 on UCMS-Induced Cognitive-Like Behavior using Novel Object Recognition (NOR) Test; Supplementary S3: Statistical Analysis Effect of AYW-KK-04 on UCMS-Induced Cognitive-Like Behavior using Morris Water Maze (MWM) test; Supplementary S4: Statistical Analysis Effect of AYW-KK-04 on UCMS-Changed Lipid Peroxidation in Hippocampus and Frontal Cortex; Supplementary S5: Statistical Analysis Effect of AYW-KK-04 on UCMS- Changed Antioxidant Enzyme Activities in Hippocampus and Frontal Cortex; Supplementary S6. Statistical Analysis Effect of AYW-KK-04 on UCMS- Changed gene expression in Hippocampus and Frontal Cortex. Supplementary S7. Validation result of the HPLC method for the determination of ellagic acid and piperine.

## Abbreviations

The following abbreviations are used in this manuscript:

ABTS	2,2′-azinobis-(3-ethylbenzothiazoline-6-sulfonic acid)
AYW-KK-04	Aayu-Wattana-Khon Kaen-04
BDNF	Brain-Derived Neurotrophic Factor
CAT	Catalase
CREB	Cyclic AMP Response Element-Binding protein
GAPDH	Glyceraldehyde-3-phosphate dehydrogenase
HPA	Hypothalamic–Pituitary–Adrenal
ICR	Institute of Cancer Research
Keap1	Kelch-like ECH-associated protein 1
LOD	Limit of detection
LOQ	Limit of quantification
MDA	Malondialdehyde
Nrf2	Nuclear factor erythroid 2-related factor 2
ROS	Reactive oxygen species
SCMC	Sodium carboxymethyl cellulose
SOD	Superoxide dismutase
TBARs	Thiobarbituric acid reactive substances
UCMS	Unpredictable Chronic Mild Stress

## References

[1] Eachus H., Ryu S. (2024). Glucocorticoid effects on the brain: From adaptive developmental plasticity to allostatic overload. J. Exp. Biol..

[2] Feng X., Jia M., Cai M., Zhu T., Hashimoto K., Yang J.J. (2025). Central-peripheral neuroimmune dynamics in psychological stress and depression: Insights from current research. Mol. Psychiatry.

[3] Choi G.E., Han H.J. (2021). Glucocorticoid impairs mitochondrial quality control in neurons. Neurobiol. Dis..

[4] Boas S.M., Joyce K.L., Cowell R.M. (2022). The NRF2-dependent transcriptional regulation of antioxidant defense pathways: Relevance for cell type-specific vulnerability to neurodegeneration and therapeutic intervention. Antioxidants.

[5] Borges de Souza P., Rodrigues A.L.S., De Felice F.G. (2025). Shared mechanisms in dementia and depression: The modulatory role of physical exercise. J. Neurochem..

[6] Ring M. (2025). An Integrative approach to HPA axis dysfunction: From recognition to recovery. Am. J. Med..

[7] Malekijahan F., Razavi S.H., Nouri M., Shafiepour M., Afraei M. (2025). Unlocking nature’s potential: The power of adaptogens in enhancing modern health and wellness. J. Agric. Food Res..

[8] Panossian A., Wikman G. (2010). Effects of adaptogens on the central nervous system and the molecular mechanisms associated with their stress-protective activity. Pharmaceuticals.

[9] Panossian A. (2017). Understanding adaptogenic activity: Specificity of the pharmacological action of adaptogens and other phytochemicals. Ann. N. Y. Acad. Sci..

[10] Tantiwongsekunakorn A., Booranasubkajorn S., Chaopeerapong T., Apichartvorakit A., Akarasereenont P. (2025). Pulse diagnosis in Thai traditional medicine: Comparing Tri-Dhātu characteristics in each innate body element between experts and device. J. Health Res..

[11] Tawee L., Uapong J. (2014). Thai Traditional Medicine in the Faculty of Medicine Siriraj Hospital.

[12] Srikumar R., Parthasarathy N.J., Manikandan S., Narayanan G.S., Sheeladevi R. (2006). Effect of Triphala on oxidative stress and on cell-mediated immune response against noise stress in rats. Mol. Cell. Biochem..

[13] Phetkate P., Kummalue T., U-pratya Y., Kietinun S. (2012). Significant increase in cytotoxic T lymphocytes and natural killer cells by Triphala: A clinical phase I study. Evid. Based Complement. Altern. Med..

[14] Horani A., Shoseyov D., Ginsburg I., Mruwat R., Doron S., Amer J., Safadi R. (2012). Triphala (PADMA) extract alleviates bronchial hyperreactivity in a mouse model through liver and spleen immune modulation and increased anti-oxidative effects. Ther. Adv. Respir. Dis..

[15] Prananda A.T., Dalimunthe A., Harahap U., Simanjuntak Y., Peronika E., Karosekali N.E., Hasibuan P.A.Z., Syahputra R.A., Situmorang P.C., Nurkolis F. (2023). *Phyllanthus emblica*: A comprehensive review of its phytochemical composition and pharmacological properties. Front. Pharmacol..

[16] Gao H., Lu H., Fang N., Su J., Li R., Wang W., Zhang Y. (2024). The potential of *Terminalia chebulain* alleviating mild cognitive impairment: A review. Front. Pharmacol..

[17] Rajaduraivelpandian S., Suresh S., Kumar R.S., Gopalakrishnan V.K. (2014). Cognitive-Enhancing Properties of the Aqueous Extract of *Terminalia bellirica* Fruits in Experimental Animal Models. Biomed. Pharmacol. J..

[18] Arcusa R., Villaño D., Marhuenda J., Cano M., Cerdà B., Zafrilla P. (2022). Potential Role of Ginger (Zingiber officinale Roscoe) in the Prevention of Neurodegenerative Diseases. Front. Nutr..

[19] Nutmakul T., Chewchinda S. (2023). Synergistic effect of Trikatuk, a traditional Thai formulation, on antioxidant and alpha-glucosidase inhibitory activities. Heliyon.

[20] Zhu H., Yan Y., Jiang Y., Meng X. (2022). Ellagic acid and its anti-aging effects on central nervous system. Int. J. Mol. Sci..

[21] Chen H., Jia H., Wang W., Cai Q., Sun J., Cui C. (2025). Polyphenol-functionalized biosensors for real-time monitoring of oxidative stress and inflammation. Ind. Crops Prod..

[22] Tripathi A.K., Ray A.K., Mishra S.K. (2022). Molecular and pharmacological aspects of piperine as a potential molecule for disease prevention and management: Evidence from clinical trials. Beni-Suef Univ. J. Basic Appl. Sci..

[23] Masraksa W., Daodee S., Monthakantirat O., Boonyarat C., Khamphukdee C., Kwankhao P., Mading A., Muenhong P., Maneenet J., Awale S. (2025). Suk-SaiYasna remedy, a traditional Thai medicine, mitigates stress-induced cognitive impairment via Keap1-Nrf2 pathway. Int. J. Mol. Sci..

[24] Maneenet J., Chotritthirong Y., Omar A.M., Choonong R., Daodee S., Monthakantirat O., Khamphukdee C., Pitiporn S., Awale S., Matsumoto K. (2025). *Nelumbo nucifera* petals ameliorate depressive-like symptom and cognitive deficit in unpredictable chronic mild stress mouse model. Nutrients.

[25] Zhu S., Wang J., Zhang Y., Li V., Kong J., He J., Li X.-M. (2014). Unpredictable chronic mild stress induces anxiety and depression-like behaviors and inactivates AMP-activated protein kinase in mice. Brain Res..

[26] Willner P. (2017). The chronic mild stress (CMS) model of depression: History, evaluation and usage. Neurobiol. Stress.

[27] Song L., Che W., Min-Wei W., Murakami Y., Matsumoto K. (2006). Impairment of the spatial learning and memory induced by learned helplessness and chronic mild stress. Pharmacol. Biochem. Behav..

[28] Ulasov A.V., Rosenkranz A.A., Georgiev G.P., Sobolev A.S. (2022). Nrf2/Keap1/ARE signaling: Towards specific regulation. Life Sci..

[29] Yang X., Liu Y., Cao J., Wu C., Tang L., Bian W., Chen Y., Yu L., Wu Y., Li S. (2025). Targeting epigenetic and post-translational modifications of NRF2: Key regulatory factors in disease treatment. Cell Death Discov..

[30] Ying Z., Fu S., Yang Y. (2025). Signaling and scavenging: Unraveling the complex network of antioxidant enzyme regulation in plant cold adaptation. Plant Stress.

[31] Liu S., Liu J., Wang Y., Deng F., Deng Z. (2025). Oxidative stress: Signaling pathways, biological functions, and disease. MedComm.

[32] Abdul Manap A.S., Sivapragasam G. (2025). Perspective on the oxidative stress biomarkers and antioxidant defense strategies. Curr. Tradit. Med..

[33] Intatham S., Taychaworaditsakul W., Khonsung P., Chansakaow S., Jaijoy K., Lertprasertsuke N., Soonthornchareonnon N., Sireeratawong S. (2024). Safety Evaluation for Acute and Chronic Oral Toxicity of Maha Pigut Triphala Containing Three Medicinal Fruits in Sprague–Dawley Rats. Biology.

[34] Chanda D., Shanker K., Pal A., Luqman S., Bawankule D.U., Mani D., Darokar M.P. (2009). Safety Evaluation of Trikatu, a Generic Ayurvedic Medicine, in Charles Foster Rats. J. Toxicol. Sci..

[35] Syed S.B., Arya H., Fu I.H., Yeh T.K., Periyasamy L., Hsieh H.P., Coumar M.S. (2017). Targeting P-glycoprotein: Investigation of piperine analogs for overcoming drug resistance in cancer. Sci. Rep..

[36] Limsakul S., Monthakantirat O., Chulikhit Y., Maneenet J., Khamphukdee C., Chotritthirong Y., Phasomsap A., Boonyarat C., Daodee S. (2024). Optimizing extraction, evaluating antioxidant activity, and analyzing bioactive compounds in Trikaysornmas formula. Adv. Pharmacol. Pharm. Sci..

[37] Bibi Sadeer N., Montesano D., Albrizio S., Zengin G., Mahomoodally M.F. (2020). The Versatility of antioxidant assays in food science and safety—Chemistry, applications, strengths, and limitations. Antioxidants.

[38] Chotritthirong Y., Sumanont Y., Daodee S., Mading A., Boonyarat C., Khamphukdee C., Kumla D., Maneenet J., Matsumoto K., Kijjoa A. (2025). Antidepressant-like effects of *Garcinia nigrolineata* resin extract in a chronic mild stress mouse model: Modulation of monoaminergic and HPA-axis pathways. Plants.

[39] Sultana R., Perluigi M., Butterfield D.A. (2013). Lipid peroxidation triggers neurodegeneration: A redox proteomics view into the Alzheimer disease brain. Free Radic. Biol. Med..

[40] Jomova K., Raptova R., Alomar S.Y., Alwasel S.H., Nepovimova E., Kuca K., Valko M. (2023). Reactive oxygen species, toxicity, oxidative stress, and antioxidants: Chronic diseases and aging. Arch. Toxicol..

[41] Chulikhit Y., Sukhano W., Daodee S., Putalun W., Wongpradit R., Khamphukdee C., Umehara K., Noguchi H., Matsumoto K., Monthakantirat O. (2021). Effects of *Pueraria candollei* Var *mirifica* (Airy Shaw and Suvat.) Niyomdham on ovariectomy-induced cognitive impairment and oxidative stress in the mouse brain. Molecules.

