# Supplementary material for: Adaptogenic and Neuroprotective Effects of the Thai Herbal Formula AYW-KK-04 Against Chronic Stress-Induced Cognitive Impairment
Source: Pharmaceuticals (Basel). 2026 Feb 21;19(2):339. doi: 10.3390/ph19020339 (PMC12944196; doi:10.3390/ph19020339)
Supplement: Supplementary file 1 [file pharmaceuticals-19-00339-s001.zip › pharmaceuticals-4142033-supplementary.pdf]

Supplementary Materials Information

Adaptogenic and Neuroprotective Effects of the Thai Herbal Formula AYW-KK-04 Against Chronic Stress-Induced Cognitive Impairment

Pathomporn Saisud [1], Orawan Monthakantirat [2], Prathan Luecha [3], Suppachai Tiyaworanant [3], Abdulwaris Mading [1], Yutthana Chotritthirong [1], Sunanthra Ruangrit [1], Nawarat Jintanamaneerat [2], Jarurat Trakanchan [2], Juthamart Maneenet [4], Suresh Awale [4], and Yaowared Sumanont [2,*]

[1] Graduate School of Pharmaceutical Sciences, Khon Kaen University, 40002, Khon Kaen, Thailand; pat-thomporn.s@kkumail.com (P.S.); abdulwaris.m@kkumail.com (A.M.); yutthana_ch@kkumail.com (Y.C.); sunansa.r@kkumail.com (S.R.)
[2] Division of Pharmaceutical Chemistry, Faculty of Pharmaceutical Sciences, 40002, Khon Kaen University, Khon Kaen, Thailandoramon@kku.ac.th (O.M.); nawaratji@kkumail.com (N.J.); jarurat_t@kkumail.com (J.T.); yaosum@kku.ac.th (Y.S.)
[3] Division of Pharmacognosy and Toxicology, Faculty of Pharmaceutical Sciences, Khon Kaen University, 40002, Khon Kaen, Thailand; prathanl@kku.ac.th (P.L.); suptiy@kku.ac.th (S.T.)
[4] Natural Drug Discovery Laboratory, Institute of Natural Medicine, University of Toyama, 2630 Sugitani, Toyama 930-0194, Japan; juthamar@inm.u-toyama.ac.jp (J.M.); suresh@inm.u-toyama.ac.jp (S.A.)
* Correspondence: yaosum@kku.ac.th (Y.S.); Tel.: +66-81-380-2357

S1. Statistical Analysis Effect of AYW-KK-04 on UCMS-Induced Cognitive-Like Behavior using the Y-Maze Test.

Table S1 One-way analysis of variance (ANOVA) test of the Y-maze test.

**Group comparison**	**Statistical analysis**	
	P	**F ($DF_{between\ group}$, $DF_{residual}$)**
Non-stress vs. UCMS + vehicle group (t-Test)	0.002	t (18) = 3.653
ANOVA followed by Tukey's post hoc test		
All group	0.002	F (3,36) = 6.302
UCMS + vehicle group vs. UCMS + Vitamin E	0.025	
UCMS + vehicle group vs. UCMS + AYW200	0.978	
UCMS + vehicle group vs. UCMS + AYW800	0.007	
UCMS + AYW200 vs. UCMS + AYW800	0.019	

S2. Statistical Analysis Effect of AYW-KK-04 on UCMS-Induced Cognitive-Like Behavior using Novel Object Recognition (NOR) Test.

Table S2 One-way analysis of variance (ANOVA) test of the NOR test.

**Group comparison**	**Statistical analysis**	
	P	**F ($DF_{between\ group}$, $DF_{residual}$)**
Non-stress vs. UCMS + vehicle group (t-Test)	< 0.001	t (18) = 11.513
ANOVA followed by Tukey's post hoc test		
All group	< 0.001	F (3,36) = 12.313
UCMS + vehicle group vs. UCMS + Vitamin E	< 0.001	
UCMS + vehicle group vs. UCMS + AYW200	0.245	
UCMS + vehicle group vs. UCMS + AYW800	< 0.001	
UCMS + AYW200 vs. UCMS + AYW800	0.012	

S3. Statistical Analysis Effect of AYW-KK-04 on UCMS-Induced Cognitive-Like Behavior using Morris Water Maze (MWM) test.

Table S3.1 One-way repeated measurement ANOVA test of MWM test on the training phase, day 1

**Group comparison**	**Statistical analysis**	
	P	**F ($DF_{between\ group}$, $DF_{residual}$)**
Non-stress vs. UCMS + vehicle group (t-Test)	0.196	t (18) = -1.343
All UCMA-induced groups	1.001	F (3,36) = 0.403

Table S3.2 One-way repeated measurement ANOVA test of MWM test on the training phase, day 2

**Group comparison**	**Statistical analysis**	
	P	**F ($DF_{between\ group}$, $DF_{residual}$)**
Non-stress vs. UCMS + vehicle group (t-Test)	< 0.001	t (18) = -10.497
ANOVA followed by Tukey's post hoc test		
All group	< 0.001	F (3,36) = 11.188
UCMS + vehicle group vs. UCMS + Vitamin E	< 0.001	
UCMS + vehicle group vs. UCMS + AYW200	0.003	
UCMS + vehicle group vs. UCMS + AYW800	< 0.001	
UCMS + AYW200 vs. UCMS + AYW800	0.873	

Table S3.3 One-way repeated measurement ANOVA test of MWM test on the training phase, day 3

**Group comparison**	**Statistical analysis**	
	P	**F ($DF_{between\ group}$, $DF_{residual}$)**
Non-stress vs. UCMS + vehicle group (t-Test)	< 0.001	t (18) = -16.745
ANOVA followed by Tukey's post hoc test		
All group	< 0.001	F (3,36) = 16.640
UCMS + vehicle group vs. UCMS + Vitamin E	< 0.001	
UCMS + vehicle group vs. UCMS + AYW200	< 0.001	
UCMS + vehicle group vs. UCMS + AYW800	< 0.001	
UCMS + AYW200 vs. UCMS + AYW800	0.823	

Table S3.4 One-way repeated measurement ANOVA test of MWM test on the training phase, day 4

**Group comparison**	**Statistical analysis**	
	P	**F ($DF_{between\ group}$, $DF_{residual}$)**
Non-stress vs. UCMS + vehicle group (t-Test)	< 0.001	t (18) = -11.953
ANOVA followed by Tukey's post hoc test		
All group	< 0.001	F (3,36) = 47.691
UCMS + vehicle group vs. UCMS + Vitamin E	< 0.001	
UCMS + vehicle group vs. UCMS + AYW200	< 0.001	
UCMS + vehicle group vs. UCMS + AYW800	< 0.001	
UCMS + AYW200 vs. UCMS + AYW800	0.944	

Table S3.5 One-way repeated measurement ANOVA test of MWM test on the training phase, day 5

**Group comparison**	**Statistical analysis**	
	P	**F ($DF_{between\ group}$, $DF_{residual}$)**
Non-stress vs. UCMS + vehicle group (t-Test)	< 0.001	t (18) = -17.747
ANOVA followed by Tukey's post hoc test		
All group	< 0.001	F (3,36) = 32.024
UCMS + vehicle group vs. UCMS + Vitamin E	< 0.001	
UCMS + vehicle group vs. UCMS + AYW200	< 0.001	
UCMS + vehicle group vs. UCMS + AYW800	< 0.001	
UCMS + AYW200 vs. UCMS + AYW800	0.910	

Table S3.6 One-way analysis of variance (ANOVA) test of MWM test on test day

Group comparison	Statistical analysis	
	P	**F ($DF_{between\ group}$, $DF_{residual}$)**
Non-stress vs. UCMS + vehicle group (t-Test)	< 0.001	t (18) = 8.498
ANOVA followed by Tukey's post hoc test		
All group	< 0.001	F (3,36) = 29.642
UCMS + vehicle group vs. UCMS + Vitamin E	< 0.001	
UCMS + vehicle group vs. UCMS + AYW200	< 0.001	
UCMS + vehicle group vs. UCMS + AYW800	< 0.001	
UCMS + AYW200 vs. UCMS + AYW800	< 0.001	

S4. Statistical Analysis Effect of AYW-KK-04 on UCMS-Changed Lipid Peroxidation in Hippocampus and Frontal Cortex.

Table S4.1 One-way analysis of variance (ANOVA) test of lipid peroxidation in hippocampus.

Group comparison	Statistical analysis	
	P	**F ($DF_{between\ group}$, $DF_{residual}$)**
Non-stress vs. UCMS + vehicle group (t-Test)	< 0.001	t (18) = -7.895
ANOVA followed by Tukey's post hoc test		
All group	< 0.001	F (3,16) = 15.527
UCMS + vehicle group vs. UCMS + Vitamin E	< 0.001	
UCMS + vehicle group vs. UCMS + AYW200	0.354	
UCMS + vehicle group vs. UCMS + AYW800	< 0.001	
UCMS + AYW200 vs. UCMS + AYW800	0.009	

Table S4.2 One-way analysis of variance (ANOVA) test of lipid peroxidation in frontal cortex.

Group comparison	Statistical analysis	
	P	**F ($DF_{between\ group}$, $DF_{residual}$)**
Non-stress vs. UCMS + vehicle group (t-Test)	< 0.001	t (18) = -9.012
ANOVA followed by Tukey's post hoc test		
All group	< 0.001	F (3,16) = 62.011
UCMS + vehicle group vs. UCMS + Vitamin E	< 0.001	
UCMS + vehicle group vs. UCMS + AYW200	0.952	
UCMS + vehicle group vs. UCMS + AYW800	< 0.001	
UCMS + AYW200 vs. UCMS + AYW800	< 0.001	

S5. Statistical Analysis Effect of AYW-KK-04 on UCMS- Changed Antioxidant Enzyme Activities in Hippocampus and Frontal Cortex.

Table S5.1 One-way analysis of variance (ANOVA) test of SOD activity in hippocampus.

Group comparison	Statistical analysis	
	P	**F ($DF_{between\ group}$, $DF_{residual}$)**
Non-stress vs. UCMS + vehicle group (t-Test)	< 0.001	t (18) = 10.891
ANOVA followed by Tukey's post hoc test		
All group	< 0.001	F (3,16) = 54.840
UCMS + vehicle group vs. UCMS + Vitamin E	< 0.001	
UCMS + vehicle group vs. UCMS + AYW200	0.992	
UCMS + vehicle group vs. UCMS + AYW800	< 0.001	
UCMS + AYW200 vs. UCMS + AYW800	< 0.001	

Table S5.2 One-way analysis of variance (ANOVA) test of SOD activity in frontal cortex.

Group comparison	Statistical analysis	
	P	**F ($DF_{between\ group}$, $DF_{residual}$)**
Non-stress vs. UCMS + vehicle group (t-Test)	< 0.001	t (18) = 16.653
ANOVA followed by Tukey's post hoc test		
All group	< 0.001	F (3,16) = 32.778
UCMS + vehicle group vs. UCMS + Vitamin E	< 0.001	
UCMS + vehicle group vs. UCMS + AYW200	0.648	
UCMS + vehicle group vs. UCMS + AYW800	< 0.001	
UCMS + AYW200 vs. UCMS + AYW800	< 0.001	

Table S5.3 One-way analysis of variance (ANOVA) test of CAT activity in hippocampus.

Group comparison	Statistical analysis	
	P	**F ($DF_{between\ group}$, $DF_{residual}$)**
Non-stress vs. UCMS + vehicle group (t-Test)	< 0.001	t (18) = 72.126
ANOVA followed by Tukey's post hoc test		
All group	< 0.001	F (3,16) = 1468.889
UCMS + vehicle group vs. UCMS + Vitamin E	< 0.001	
UCMS + vehicle group vs. UCMS + AYW200	0.999	
UCMS + vehicle group vs. UCMS + AYW800	< 0.001	
UCMS + AYW200 vs. UCMS + AYW800	< 0.001	

Table S5.4 One-way analysis of variance (ANOVA) test of CAT activity in frontal cortex.

Group comparison	Statistical analysis	
	P	**F ($DF_{between\ group}$, $DF_{residual}$)**
Non-stress vs. UCMS + vehicle group (t-Test)	< 0.001	t (18) = 124.690
ANOVA followed by Tukey's post hoc test		
All group	< 0.001	F (3,16) = 3682.042
UCMS + vehicle group vs. UCMS + Vitamin E	< 0.001	
UCMS + vehicle group vs. UCMS + AYW200	0.185	
UCMS + vehicle group vs. UCMS + AYW800	< 0.001	
UCMS + AYW200 vs. UCMS + AYW800	< 0.001	

S6. Statistical Analysis Effect of AYW-KK-04 on UCMS- Changed gene expression in Hippocampus and Frontal Cortex.

Table S6.1 One-way analysis of variance (ANOVA) test of BDNF expression in hippocampus.

**Group comparison**	**Statistical analysis**	
	P	**F ($DF_{between\ group}$, $DF_{residual}$)**
Non-stress vs. UCMS + vehicle group (t-Test)	< 0.001	t (18) = 39.179
ANOVA followed by Tukey's post hoc test		
All group	< 0.001	F (3,16) = 830.278
UCMS + vehicle group vs. UCMS + Vitamin E	< 0.001	
UCMS + vehicle group vs. UCMS + AYW200	0.932	
UCMS + vehicle group vs. UCMS + AYW800	< 0.001	
UCMS + AYW200 vs. UCMS + AYW800	< 0.001	

Table S6.2 One-way analysis of variance (ANOVA) test of BDNF expression in frontal cortex.

**Group comparison**	**Statistical analysis**	
	P	**F ($DF_{between\ group}$, $DF_{residual}$)**
Non-stress vs. UCMS + vehicle group (t-Test)	< 0.001	t (18) = 40.689
ANOVA followed by Tukey's post hoc test		
All group	< 0.001	F (3,16) = 43.875
UCMS + vehicle group vs. UCMS + Vitamin E	< 0.001	
UCMS + vehicle group vs. UCMS + AYW200	0.997	
UCMS + vehicle group vs. UCMS + AYW800	< 0.001	
UCMS + AYW200 vs. UCMS + AYW800	< 0.001	

Table S6.3 One-way analysis of variance (ANOVA) test of CREB expression in hippocampus.

**Group comparison**	**Statistical analysis**	
	P	**F ($DF_{between\ group}$, $DF_{residual}$)**
Non-stress vs. UCMS + vehicle group (t-Test)	< 0.001	t (18) = 18.081
ANOVA followed by Tukey's post hoc test		
All group	< 0.001	F (3,16) = 257.279
UCMS + vehicle group vs. UCMS + Vitamin E	< 0.001	
UCMS + vehicle group vs. UCMS + AYW200	0.449	
UCMS + vehicle group vs. UCMS + AYW800	< 0.001	
UCMS + AYW200 vs. UCMS + AYW800	< 0.001	

Table S6.4 One-way analysis of variance (ANOVA) test of CREB expression in frontal cortex.

**Group comparison**	**Statistical analysis**	
	P	**F ($DF_{between\ group}$, $DF_{residual}$)**
Non-stress vs. UCMS + vehicle group (t-Test)	< 0.001	t (18) = 23.912
ANOVA followed by Tukey's post hoc test		
All group	< 0.001	F (3,16) = 76.023
UCMS + vehicle group vs. UCMS + Vitamin E	< 0.001	
UCMS + vehicle group vs. UCMS + AYW200	0.997	
UCMS + vehicle group vs. UCMS + AYW800	< 0.001	
UCMS + AYW200 vs. UCMS + AYW800	< 0.001	

Table S6.5 One-way analysis of variance (ANOVA) test of Nrf2 expression in hippocampus.

**Group comparison**	**Statistical analysis**	
	P	**F ($DF_{between\ group}$, $DF_{residual}$)**
Non-stress vs. UCMS + vehicle group (t-Test)	< 0.001	t (18) = 5.821
ANOVA followed by Tukey's post hoc test		F (3,16) = 72.980
All group	< 0.001	
UCMS + vehicle group vs. UCMS + Vitamin E	< 0.001	
UCMS + vehicle group vs. UCMS + AYW200	0.981	
UCMS + vehicle group vs. UCMS + AYW800	< 0.001	
UCMS + AYW200 vs. UCMS + AYW800	< 0.001	

Table S6.6 One-way analysis of variance (ANOVA) test of Nrf2 expression in frontal cortex.

**Group comparison**	**Statistical analysis**	
	P	**F ($DF_{between\ group}$, $DF_{residual}$)**
Non-stress vs. UCMS + vehicle group (t-Test)	< 0.001	t (18) = 10.460
ANOVA followed by Tukey's post hoc test		F (3,16) = 326.734
All group	< 0.001	
UCMS + vehicle group vs. UCMS + Vitamin E	< 0.001	
UCMS + vehicle group vs. UCMS + AYW200	1.000	
UCMS + vehicle group vs. UCMS + AYW800	< 0.001	
UCMS + AYW200 vs. UCMS + AYW800	< 0.001	

Table S6.7 One-way analysis of variance (ANOVA) test of Keap1 expression in hippocampus.

**Group comparison**	**Statistical analysis**	
	P	**F ($DF_{between\ group}$, $DF_{residual}$)**
Non-stress vs. UCMS + vehicle group (t-Test)	< 0.001	t (18) = -17.577
ANOVA followed by Tukey's post hoc test		F (3,16) = 8.612
All group	0.001	
UCMS + vehicle group vs. UCMS + Vitamin E	0.004	
UCMS + vehicle group vs. UCMS + AYW200	0.999	
UCMS + vehicle group vs. UCMS + AYW800	0.022	
UCMS + AYW200 vs. UCMS + AYW800	0.041	

Table S6.8 One-way analysis of variance (ANOVA) test of Keap1 in frontal cortex.

**Group comparison**	**Statistical analysis**	
	P	**F ($DF_{between\ group}$, $DF_{residual}$)**
Non-stress vs. UCMS + vehicle group (t-Test)	< 0.001	t (18) = -8.296
ANOVA followed by Tukey's post hoc test		F (3,16) = 9.207
All group	< 0.001	
UCMS + vehicle group vs. UCMS + Vitamin E	0.003	
UCMS + vehicle group vs. UCMS + AYW200	0.781	
UCMS + vehicle group vs. UCMS + AYW800	0.005	
UCMS + AYW200 vs. UCMS + AYW800	0.034	

S7. Validation result of the HPLC method for the determination of ellagic acid and piperine

Parameter		Ellagic acid	Piperine
Linearity	Concentration range (µg/mL)	15 - 360	50 - 1000
	Linear regression equation	y = 111.59x + 33.837	y= 20.536x + 223.94
	Correlation coefficient (R^2)	0.9999	0.9999
Sensitivity	LOD (µg/mL)	0.2	5.0
	LOQ (µg/mL)	0.25	12.0
Precision	Intra-day (%RSD)	0.22 – 1.76	0.51 – 1.03
	Inter-day (%RSD)	0.20 – 1.43	1.28 – 1.73
Accuracy	Accuracy (%recovery)	99.80 – 103.61	101.23 – 104.21
